# Giant Tophaceous Gout: The Importance of Therapeutic Compliance

**DOI:** 10.7759/cureus.54464

**Published:** 2024-02-19

**Authors:** Ricardo Silva Veiga, Ines B Mesquita, Joana Gomes da Cunha, José Pedro Mota Fonseca, Sofia Pereira

**Affiliations:** 1 Internal Medicine, Hospital de São Teotónio, Unidade Local de Saúde de Viseu Dão-Lafões, EPE, Viseu, PRT; 2 Internal Medicine, Hospital Distrital Figueira da Foz, Figueira da Foz, PRT

**Keywords:** uric acid renal dysfunction, kidney disease, arthralgias, crystal arthropathies, gout

## Abstract

Gout is a metabolic disease resulting from the deposition of monosodium urate crystals in joints, tissues, and organs. Nowadays, the treatment of hyperuricemia is easily accessible and widespread and mainly consists of xanthine oxidase inhibitors and uricosurics. In refractory and advanced cases of gout, amputation surgery may be required. The authors present the case of an 85-year-old man who is non-compliant with hypouricemic medication, has exuberant gout, and has refused amputation surgery several times. The patient went to the emergency department with a triad of acute kidney injuries, acute gout, and poorly controlled pain. Cases of tophaceus gout such as the one presented are very rare nowadays.

## Introduction

Gout is an inflammatory arthritis resulting from an excess of uric acid in the blood, causing its deposition in the joints, bones, and other areas of the body. Gout typically develops in four different phases: the asymptomatic phase, acute gouty arthritis, the intercritical period, and chronic gout. Although most patients with hyperuricemia remain asymptomatic throughout their lives, the risk of acute gouty arthritis is greater after 20 years of persistently elevated uric acid levels [1], while chronic gout occurs frequently 10 years after the onset of recurrent polyarticular gout [2]. Given that the time required for gouty tophi to appear is long, this pathology is more common in older patients. Also, it correlates with the severity of the hyperuricemia [3], the use of medications such as diuretics or aspirin [2], and inadequate treatment. Diagnosis in the advanced stages of the disease is essentially clinical. Still, it can be confirmed by aspiration of synovial fluid or gouty tophi, where monosodium urate crystals with negative birefringence are identified on optical microscopy [4].

## Case presentation

We present the case of an 85-year-old, partially dependent man with a modified Rankin score of 2, who was brought to the urgency department for vomits and arthralgia (mainly on the feet and hands). His past medical history was significant for obesity, chronic kidney disease secondary to the use of nonsteroidal anti-inflammatory drugs (NSAIDs), hemorrhagic stroke one year before (without sequelae), and hyperuricemia (known for several years with consequent gout tophi). Given the patient's known risk factors for elevated uric acid (obesity, non-hypouricemic diet), his non-compliance with hypouricemic therapy, and the highly characteristic gout tophi lesions that allow the diagnosis to be made without necessarily microscopic confirmation, the patient had previously been diagnosed for this condition by his family doctor, although it is unknown whether synovial fluid aspiration was performed at some point. He had previously been evaluated by an orthopedics specialist, who recommended limb amputation given the extent of the tophi gout, which the patient refused. The patient was also awaiting evaluation by the rheumatology specialty in an outpatient consultation. His chronic gout was previously managed with NSAIDs and febuxostat 80 mg daily, although the patient was not adherent to the hypouricemic therapy. Additionally, he took lisinopril 20 mg, amlodipine 20 mg, and chlorthalidone 50 mg daily for hypertension. He had no history of past medical allergies.

**Figure 1:**
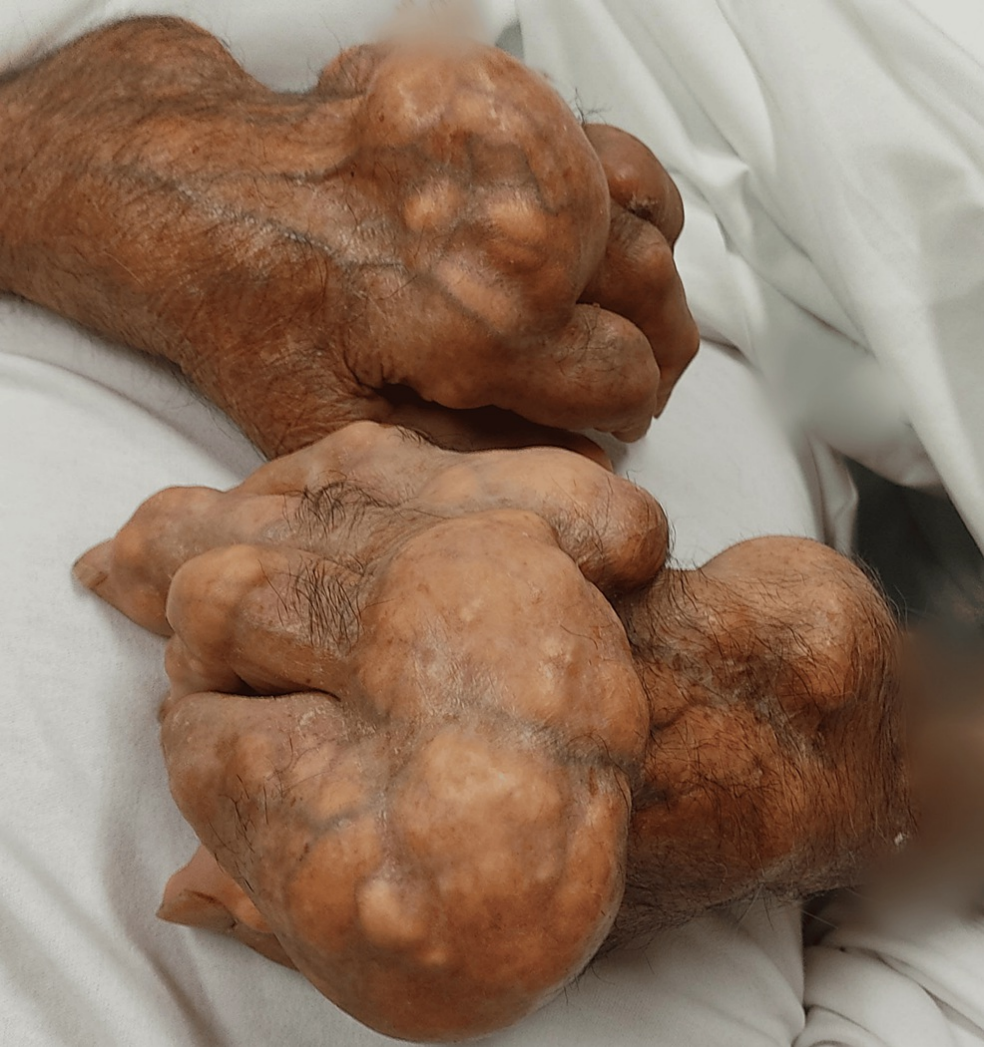
Large tophi on both hands

**Figure 2:**
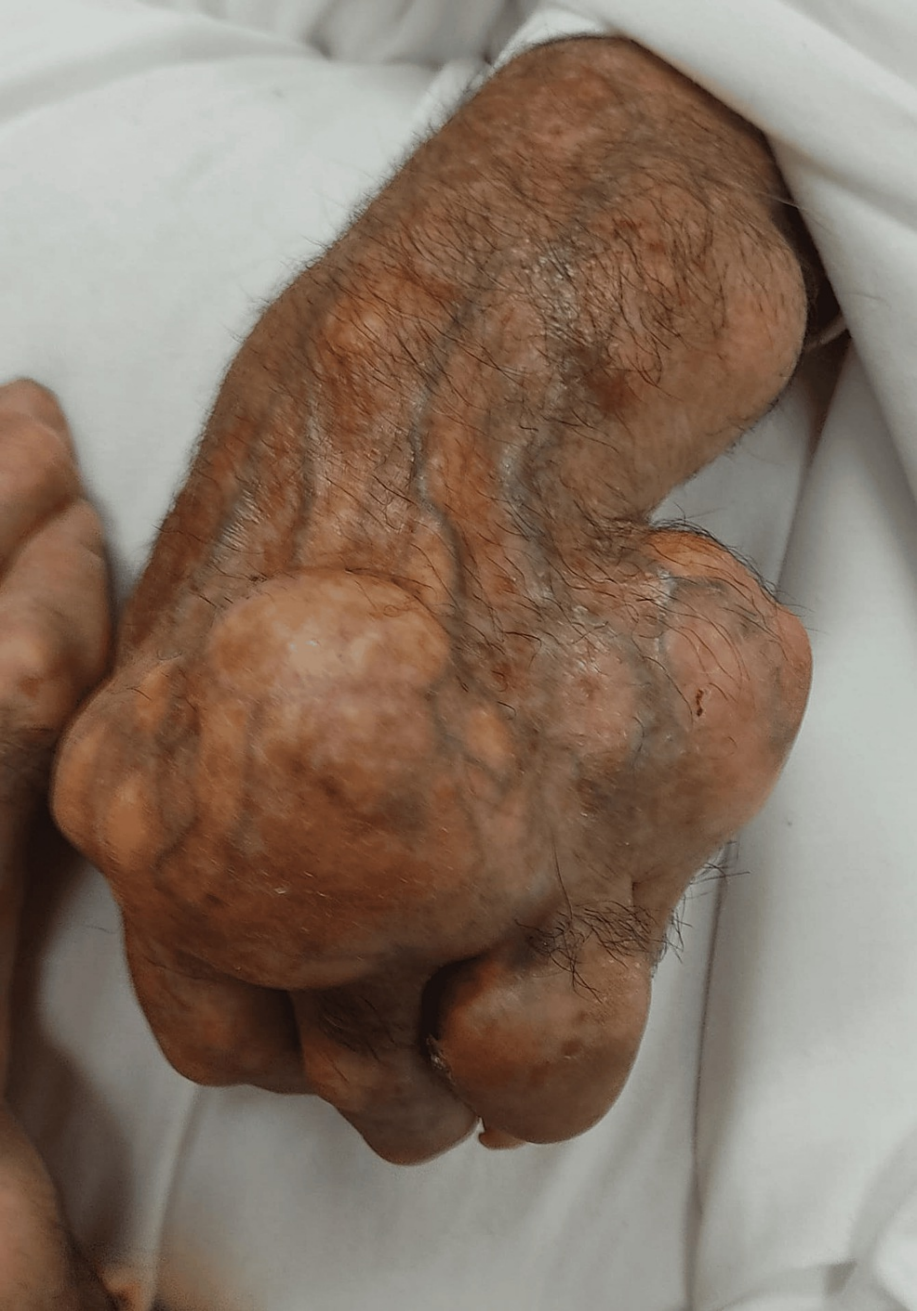
Tophi of the left-hand

**Figure 3:**
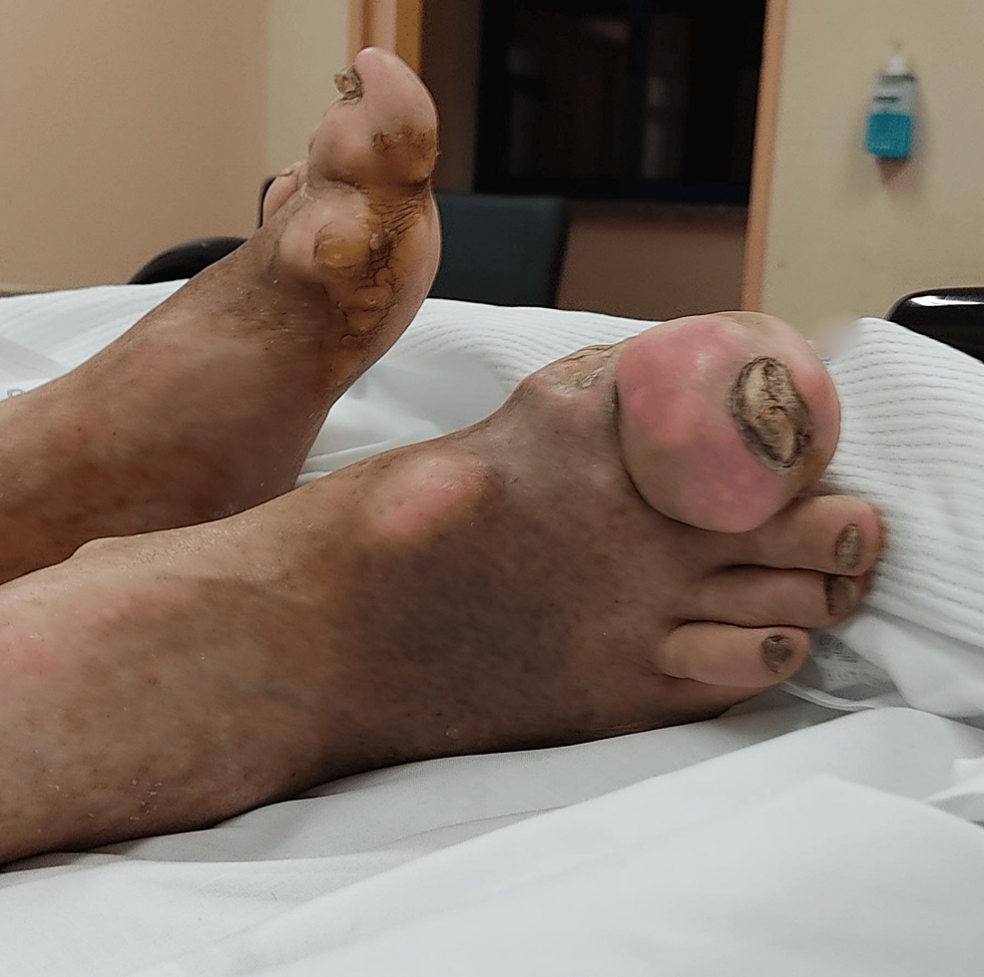
Large tophi at the first metatarsophalangeal and interphalangeal joints of the feet

At the initial evaluation, the patient was hemodynamically stable and oriented, with skin and mucose clearly dehydrated. He presented an exuberant, painful gout trophy in both hands (Figures 1-2) and feet (Figure 3), as well as in his elbows and knees with less gravity. The patient underwent an analytical study (Table 1) that revealed an increase in inflammatory parameters, with a c-reactive protein of 16.17 mg/dL, in addition to an increase in creatinine (2.6 mg/dL, for a baseline of 1.3 mg/dL) and urea (180 mg/dL). The uric acid value was also measured and was high (9.6 mg/dL). A renal ultrasound was performed, which revealed no significant findings. A urine test strip revealed leukocyturia and proteinuria. The diagnoses were assumed to be urinary tract infection, non-oliguric acute kidney injury of presumed prerenal etiology, and gouty tophi with joint deformation and poorly controlled pain.

**Table 1:** Summary of laboratory results Hb: hemoglobin; CRP: C-reactive protein

Test		First admission	Reference range
Leukocytes (/μL)		6000	4500-11500
Hb (g/dL)		11.9	14.0-18.0
Uric acid (mg/dL)		9.6	3.5-7.2
Creatinine (mg/dL)		2.6	0.6-1.3
Urea (mg/dL)		180	19-49
Sodium (mEq/L)		145	135-145
Potassium (mEq/L)		5.0	3.5-5.0
Chloride (mEq/L)		109	95-110
CRP (mg/dL)		16.17	0.0-0.5
Procalcitonin (ng/mL)		0.28	0.0-0.5

The patient was admitted for stabilization, having completed seven days of hospitalization and seven days of antibiotic therapy with ceftriaxone and fluid therapy, with resolution of the acute kidney injury. *Proteus mirabilis* was isolated in the urine, the blood culture was negative, and the control of joint pain was effective with paracetamol. Given the progressive worsening of the patient's general condition, his previous wishes, and his personal history, it was decided that he would not benefit from immediate collaboration from other specialties, so he was discharged pending a rheumatology consultation. Also, at discharge, the patient was given instructions to take febuxostat 80 mg daily, colchicine 1 mg daily, nebivolol 5 mg daily, and amlodipine 5 mg daily. He was also advised to stop the remaining therapies, namely the use of NSAIDs. Instead, he should use other analgesics like paracetamol, which proved to be effective during hospitalization.

## Discussion

The most typical initial presentation of gout is in the form of monoarthritis, with the most frequently affected site being the first metacarpophalangeal joint. In these cases, the joint must be aspirated and the fluid analyzed. Microscopy shows negatively birefringent needle-shaped monosodium urate crystals. Liquid microbiology should be requested, as well as cytology and biochemistry, to rule out other differential diagnoses, such as septic arthritis [5].

In patients without adequate treatment (xanthine oxidase inhibitors are the best choice as initial treatment in chronic gout) [6], the development of gouty tophi typically occurs after 10 years of recurrent gout. The disease is more prevalent in men and increases with age, with the fingers and toes being one of the most frequent sites of appearance [2]. In chronic gout, the joints involved can sometimes present a symmetrical pattern and can simulate other pathologies such as rheumatoid arthritis [7]. Several risk factors can trigger gout, among which genetic mutations, advanced age, family history, osteoarthritis, alcohol consumption, or thiazide diuretics stand out [8-9]. In the case presented, the contribution of chlorthalidone to the worsening of gout is worth highlighting since its role is already known [10]. In fact, Raja et al. carried out a cross-sectional, prospective study involving 330 participants on adult hypertensive patients and concluded that individuals were significantly more hyperuricaemic with significantly higher uric acid values in the group treated with thiazide diuretics [11]. The mechanism responsible for the increase in uric acid levels in patients treated with thiazide diuretics is still unknown, but it is thought that it may be due to the inhibition of the human voltage-driven drug efflux transporter, hNPT4/SLC17A3, which has the function of urate excretion at the level of the proximal renal tubules [12].

Although hyperuricemia is a very prevalent disease in the population, its treatment has proven to be effective and easily accessible for most people, and urate-lowering therapy is indicated in all patients with a diagnosis of gout, particularly in those who have recurring arthritis, tophi, take diuretics or have renal impairment. Additionally, if the patient is taking diuretics, if possible, they should be replaced with another anti-hypertensive drug, as long as the blood pressure is controlled. Diet modifications and exercise should also be advised to reduce body weight, and alcoholic drinks and high-purine foods should be reduced. For the management of acute attacks, NSAIDs or colchicine are the drugs of choice, but other factors should be considered, like co-morbidities or renal function. The urate-lowering treatment should aim initially to maintain the urate levels below 5 mg/dl to dissolve the crystals and reduce the tophi. This target can be adjusted afterward to 6 mg/dl when the patient remains free of symptoms. The urate-lowering treatment recommended as the first line is allopurinol, but febuxostat can be used as an alternative second line, especially in patients with renal impairment, which limits the escalation of allopurinol. Uricosuric agents like sulfinpyrazone or probenecid can also be an alternative in patients with renal impairment [6,13]. Surgical treatment is usually reserved for patients with ulceration, septicemia, or joint deformation and ranges from enucleation to amputation [5].

## Conclusions

Cases like the one presented are nowadays extremely rare, even more so because this is a patient who refused surgery until the end of his life, which, combined with his refusal to take hypouricemia medication, led to the evolution of so many significant tophaceous lesions. This case also illustrates the real challenge in the management of the triad of kidney disease, chronic gout with periodic exacerbations, and pain control of the arthralgias caused by the gouty tophy. The differential diagnosis must be made with other crystal arthropathies, infectious arthritis (especially if monoarthritis), and with other chronic polyarthritis.
